# Current status of endovascular treatment for older adults with acute large vessel occlusion stroke in China: subgroup analysis of ANGEL act registry

**DOI:** 10.3389/fneur.2023.1114556

**Published:** 2023-04-18

**Authors:** Bin Han, Dapeng Sun, Raynald, Baixue Jia, Xu Tong, Anxin Wang, Dapeng Mo, Feng Gao, Ning Ma, Zhongrong Miao

**Affiliations:** ^1^Shanxi Key Laboratory of Brain Disease Control, Department of Neurology, Shanxi Provincial People’s Hospital, Taiyuan, China; ^2^Department of Interventional Neuroradiology, Beijing Tiantan Hospital, Capital Medical University, Beijing, China; ^3^Department of Neurology, Beijing Tiantan Hospital, Capital Medical University, Beijing, China; ^4^China National Clinical Research Center for Neurological Diseases, Beijing Tiantan Hospital, Beijing, China

**Keywords:** endovascular treatment, large vessel occlusion, older adults, safety and efficacy, clinical outcome

## Abstract

**Background:**

Although endovascular treatment (EVT) has become the standard treatment for acute large vessel occlusion (LVO), its safety and efficacy in older adults have not been fully determined. The present study aimed to compare the safety and efficacy of EVT for acute LVO between younger (<80 years old) and older adults (≥80 years old) in the Chinese population.

**Methods:**

The subjects were selected from the ANGEL-ACT registry (endovascular treatment key technique and emergency workflow improvement of acute ischemic stroke). The 90-day modified Rankin score (mRS), successful recanalization, procedure duration, number of passes, intracranial hemorrhage (ICH), and mortality within 90 days were compared after adjusting for confounders.

**Results:**

A total of 1,691 patients, 1,543 classified as young and 148 classified as older, were included. We observed that young and older adults had a similar 90-day mRS distribution, successful recanalization, procedure duration, number of passes, ICH, and mortality within 90 days (all *p* > 0.05). The rate of 90-day mRS 0–3 was found to be higher in young patients than in older adults (39.9% vs. 56.5%, odds ratio = 0.64, 95% confidence interval = 0.44–0.94, *p* = 0.022).

**Conclusion:**

We found that patients less than or greater than 80 years of age share similar clinical outcomes, without increasing the risk of ICH and mortality.

## Introduction

Older age is a major factor that contributes to stroke occurrence and poor stroke outcomes (1). It is expected that the number of acute ischemic stroke cases in older adults will continue to increase owing to population growth, the global aging population, and advances in stroke treatment (2, 3). Thus, the problem of acute ischemic stroke treatment in older adults has become increasingly significant. The benefits of homogenizing mechanical thrombectomy in older adults are still controversial. A subgroup analysis of the Highly Effective Reperfusion Evaluated in Multiple Endovascular Stroke Trials (HERMES) collaboration showed that patients aged ≥ 80 years might benefit from endovascular treatment (EVT) more than from conservative treatment (4). Conversely, the NASA registry reported that an age greater than 80 years is predictive of poor clinical outcomes and increased mortality compared with younger patients (5). Ali Alawieh et al. (6) retrospectively analyzed prospective database data of patients with acute ischemic stroke (AIS) receiving EVT in seven comprehensive stroke centers in the United States. The results showed that the 90-day mortality rate was higher than in the young group. This result may be attributed to the probability of bleeding after thrombectomy in the older adult group being significantly higher than that in the young group, and the proportion of patients with good prognoses showed a downward trend.

With the available research data primarily from Western populations, the information available regarding efficacy and safety in the Asian population is lacking. Moreover, the Asian population has different features compared with the Western populations in most of the previous trials and registries, such as more prevalent intracranial atherosclerotic disease (ICAD). Recently, the prospective Chinese Endovascular Treatment Key Technique and Emergency Work Flow Improvement of Acute Ischemic Stroke (ANGEL-ACT Registry) was conducted to evaluate the utilization and subsequent outcomes of EVT-treated AIS patients. Therefore, our study aimed to evaluate the safety and efficacy of mechanical thrombectomy in older adults in China and provide real-world evidence of the effectiveness of EVT in older adults.

## Materials and methods

### Study population and design

We present a subgroup analysis of the ANGEL-ACT registry study (endovascular treatment key technique and emergency workflow improvement of acute ischemic stroke), which was a prospective multicenter, nationwide study involving 1,793 consecutive adult patients who underwent EVT at 111 hospitals in 26 provinces in China between November 2017 and March 2019.

**Figure 1:**
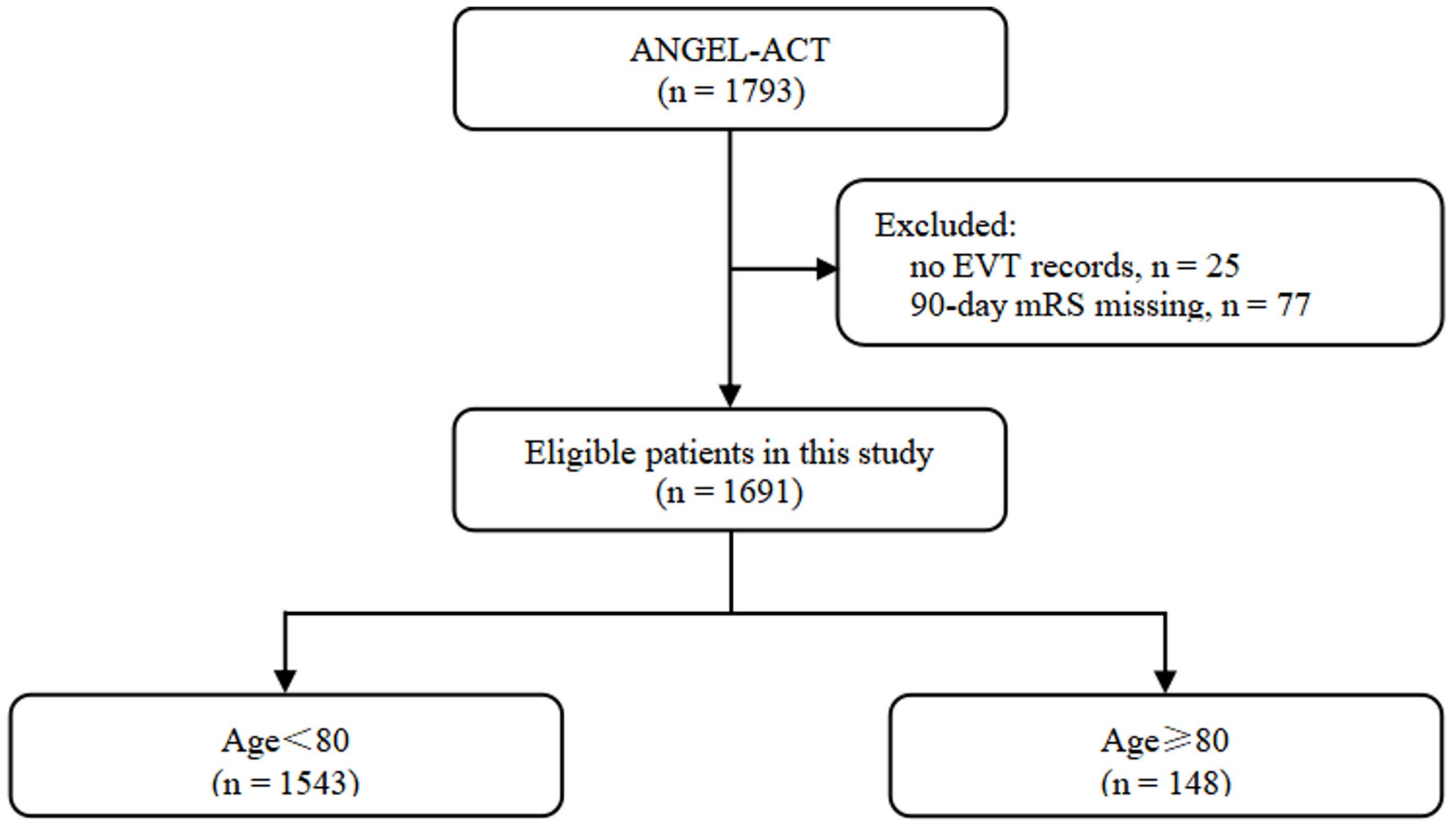
Flowchart of patient selection.

The inclusion criteria for the ANGEL-ACT registry, data collection methods, and radiological assessment have been previously described by Jia et al. (7) Of the 1,793 eligible patients, 102 were excluded due to the absence of EVT records (n = 25) and missing 90-day modified Rankin score (mRS; *n* = 77; Figure 1). The study protocol was approved by the ethics committees of each participating center. Informed consent was obtained from the participants or their legally authorized representatives prior to enrollment in the ANGEL-ACT registry.

An assessment of baseline, procedure, and outcome variables, including demographic characteristics, premorbid mRS, admission mode, medical history, admission National Institutes of Health Stroke Scale (NIHSS), imaging and laboratory results, procedure details, postprocedural management, time-metric data, and mRS after 90 days, was conducted. The NIHSS and mRS were recorded by trained and certified investigators.

Imaging data, such as the Alberta Stroke Program Early CT Score (ASPECTS) (8, 9) on baseline CT, presence of tandem occlusion, underlying intracranial atherosclerosis disease (ICAD) (10) (degree of stenosis > 70% or stenosis > 50% with distal blood flow impairment), intra-procedural complications, modified Thrombolysis in Cerebral Infarction (mTICI) (11) on digital subtraction angiography (DSA), and intracranial hemorrhage (ICH) on post-treatment imaging, were evaluated by the imaging core laboratory blinded to clinical information.

### Outcome measures

The primary outcome was the 90-day ordinal mRS distribution. The secondary outcomes included excellent outcomes, defined as a 90-day mRS of 0–1. Good functional independence was defined as a 90-day mRS of 0–2. Moderate functional independence was defined as a 90-day mRS of 0–3. Other outcomes included change in NIHSS score at 24 h and 7 days, puncture to recanalization/end of procedure time, number of MT passes, and successful recanalization at the final angiogram. The safety outcomes were death within 90 days, any ICH that occurred within 24 h, symptomatic intracranial hemorrhage (SICH) according to the Heidelberg Bleeding Classification, incidence of intraoperative distal embolism or ectopic embolism, and intraoperative complications.

### Statistical analysis

Data are presented as medians [interquartile ranges (IQRs)] for continuous and ordinal variables and percentages for categorical variables. We compared baseline characteristics between the two groups using the Mann–Whitney *U*-test for continuous and ordinal variables and Pearson’s chi-square test or Fisher’s exact test for categorical variables. The adjusted odds ratio (OR), common OR, and 95% confidence intervals (CIs) were calculated using a binary or ordinal logistic regression model or generalized linear model, respectively. All baseline variables with a significant difference of *p* < 0.05 as potential confounders were adjusted.

In addition to conventional multivariate analysis, propensity scores were constructed for adjustment (propensity scores were derived using a logistic regression model that included all baseline variables for the first-line treatment regimen).

Treatment effect modification of the primary outcome was explored in the following subgroups: sex (male vs. female), prior intravenous thrombolysis, stroke subtype according to the Trial of ORG 10172 in Acute Stroke Treatment (TOAST) criteria (large artery atherosclerosis versus cardioembolism vs. other or unknown etiology), occlusion circulation (anterior circulation vs. posterior circulation), underlying ICAD, and tandem lesions. Treatment effect size heterogeneity across subgroups was tested by including the corresponding multiplicative interaction term in the logistic regression model. All analyses were conducted using the SAS software (version 9.4: SAS Institute Inc., Cary, NC, United States). Statistical significance was set at a two-sided *p*-value of <0.05.

## Results

### Baseline characteristics

Of the 1,793 patients enrolled in the ANGEL-ACT study, 1,691 were included in the analysis. There were 1,543 (91.2%) aged < 80 years and 148 (8.8%) aged ≥ 80 years. A summary of the baseline clinical and imaging characteristics of the overall cohort of patients receiving EVT, stratified by age, is provided in Table 1. Patients aged ≥ 80 years in the study cohort had a higher incidence of coronary heart disease (25% vs. 14.1%, *p* < 0.001) and atrial fibrillation history (59.5% vs. 28.4%, *p* < 0.001) and higher NIHSS scores on admission (18 vs.16, *p* < 0.001). The prevalence of underlying ICAD was lower in the ≥80 group (12.2% vs. 31.8%, *p* < 0.001). We also observed that the use of balloon angioplasty (7.4% vs. 23.7%, *p* < 0.001), stenting (7.4% vs. 19.8%, *p* < 0.001), and GPIIb/IIIa inhibitors (36.5% vs. 53.5%, *p* < 0.001) was lower in this group. Consistently, the onset to puncture time was shorter in this group (251 min vs. 305 min, *p* < 0.001). No significant differences were observed between the two groups in the remaining baseline variables.

**Table 1:** Baseline characteristics of patients undergoing thrombectomy with Age < 80 vs. Age ≥ 80 years.

Baseline and procedure variables	Age < 80 years (*n* = 1,543)	Age ≥ 80 years (*n* = 148)	*p*-value
Male sex	1,059(68.6)	66(44.6)	<0.001
History of hypertension	876(56.8)	91(61.5)	0.268
History of diabetes mellitus	282(18.3)	25(16.9)	0.677
History of dyslipidemia	143(9.3)	11(7.4)	0.459
History of coronary heart disease	218(14.1)	37(25.0)	<0.001
History of atrial fibrillation	438(28.4)	88(59.5)	<0.001
History of stroke	337(21.8)	44(29.7)	0.028
Cigarette smoking			<0.001
Never smoker	878(56.90)	124(83.78)	
Ex-smoker	552(35.8)	9(6.1)	
Current smoker	113(7.3)	15(10.1)	
Systolic blood pressure, median (IQR), mmHg	145(130–160)	150(136–165)	0.062
NIHSS score, median (IQR)	16(11–21)	18(14–23)	<0.001
ASPECTS, median (IQR)^a^	9(7–10)	10(7–10)	0.076
Premorbid mRS			0.067
0	1,341(86.96)	119(80.41)	
1	173(11.22)	26(17.57)	
2	28(1.82)	3(2.03)	
Occlusion circulation			0.184
Anterior circulation	1,199(77.7)	122(82.4)	
Posterior circulation	344(22.3)	26(17.6)	
Occlusion site			0.903
Internal carotid artery	391(25.3)	37(25.0)	
Middle cerebral artery M1 segment	671(43.5)	66(44.6)	
Vertebro-basilar artery	5(0.3)	1(0.7)	
Other intracranial arteries	476(30.9)	44(29.7)	
Underlying ICAD	490(31.8)	18(12.2)	<0.001
Stroke subtype by TOAST criteria			<0.001
Large artery atherosclerosis	804(52.1)	50(33.8)	
Cardioembolism	481(31.2)	83(56.1)	
Other or unknown etiology	189(12.2)	7(4.7)	
Prior use of antiplatelet agents	257(16.7)	25(16.7)	0.941
Prior use of anticoagulants	61(4.0)	8(5.4)	0.394
Type of anesthesia			0.531
Local anesthesia only	663(42.8)	69(46.6)	
Local anesthesia plus sedation	622(40.3)	59(39.9)	
General anesthesia	258(16.7)	20(13.5)	
Stentriever-first techniques	1,342(87.0)	137(92.6)	0.050
Aspiration-first	311(20.2)	31(21.0)	0.820
Intra-arterial thrombolysis	134(8.7)	10(6.8)	0.422
Balloon angioplasty	365(23.7)	11(7.4)	<0.001
Stenting	306(19.8)	11(7.4)	<0.001
Intra-procedural use of heparin	768(49.8)	69(46.6)	0.464
Intra-procedural use of GPIIb/IIIa	825(53.5)	54(36.5)	<0.001
Door-to-puncture time, median (IQR), min	120(80–179)	131(90–182)	0.4348
Onset-to-puncture time, median (IQR), min	305(215–445)	251(198–375)	<0.001

Values are numbers with percentages in parentheses, unless indicated otherwise. ^a^ASPECTS for anterior circulation stroke; and pc-ASPECTS for posterior circulation stroke. ASPECTS, Alberta stroke program early CT score; ICAD, intracranial atherosclerotic disease; IQR, interquartile range; NIHSS, National Institutes of Health Stroke Scale; pc-ASPECTS, posterior circulation Alberta stroke program early CT score; TOAST, Trial of ORG 10172 in Acute Stroke Treatment.

### Outcome measures

**Figure 2:**
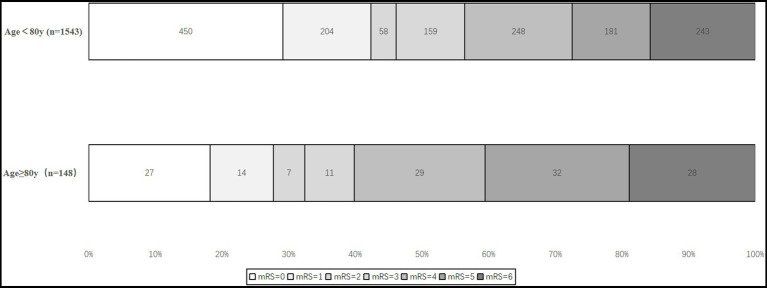
Shift on 90-day modified Rankin scale (mRS) score of age < 80 years vs. age ≥ 80 years.

Table 2 provides the primary, secondary, and safety outcome comparisons between the patients aged < 80 and ≥ 80 years. Before adjusting for confounders, we found that age ≥ 80 years was associated with a shift toward worse outcomes (4 (1–5) vs. 3[0–5], common OR 0.58, 95% CI 0.43–0.78, *p* < 0.001), lower odds of an excellent outcome (27.7% vs. 42.4%, OR 0.52, 95% CI 0.36–0.76, *p* < 0.001), good functional independence (32.4% vs. 46.1%, OR 0.56, 95% CI 0.39–0.80, *p* < 0.001), and moderate functional independence (39.9% vs. 56.5%, OR 0.51, 95% CI 0.36–0.72, *p* < 0.001). We did not find significant differences in mortality within 90 days (18.9% vs. 15.6%, OR 1.25, 95% CI 0.81–1.93, *p* = 0.316). In the adjusted analysis, there was no difference in the 90-day mRS ordinal shift (Figure 2), excellent outcome, and good functional independence between ages < 80 and ≥ 80 years (*p* > 0.05). However, an age < 80 years was associated with a higher rate of moderate functional independence (OR 0.64, 95% CI 0.44–0.94, *p* < 0.022). The safety outcomes were similar in patients aged < 80 years and patients aged ≥ 80 years (any ICH: 21.81% vs. 25.3%, *p* = 0.724; SICH: 6.97% vs. 7.6%, *p* = 0.954; incidence of intraoperative distal embolism or ectopic embolism: 4.86% vs. 6.8%, *p* = 0.996; and intraoperative complications: 86.65%, 0.7%, vs. 85.14%, *p* = 0.857).

**Table 2:** Outcome measures of patients undergoing thrombectomy with Age < 80 versus Age ≥ 80 years.

Outcome variables	Age < 80 years	Age ≥ 80 years	Unadjusted analysis		Adjusted model 1^a^		Adjusted model 2^b^	
			Effect size (95% CI)	*p*-value	Effect size (95% CI)	*p*-value	Effect size (95% CI)	*p*-value
**Primary outcome**								
mRS at 90 days, median (IQR)	3(0–5)	4(1–5)	0.58(0.43–0.78)^c^	<0.001	0.74(0.54–1.02)^c^	0.065	0.75(0.55–1.03)^c^	0.074
**Secondary outcomes**								
mRS 0–1 at 90 days	654(42.4)	41(27.7)	0.52(0.36–0.76)^d^	<0.001	0.68(0.45–1.02)^d^	0.060	0.68(0.46–1.01)^d^	0.053
mRS 0–2 at 90 days	712(46.1)	48(32.4)	0.56(0.39–0.80)^d^	<0.001	0.73(0.49–1.08)^d^	0.113	0.73(0.50–1.07)^d^	0.103
mRS 0–3 at 90 days	871(56.5)	59(39.9)	0.51(0.36–0.72)^d^	<0.001	0.64(0.44–0.94)^d^	0.022	0.66(0.46–0.95)^d^	0.024
Death within 90 days	243(15.6)	28(18.9)	1.25(0.81–1.93)^d^	0.316	0.90(0.54–1.50)^d^	0.675	0.93(0.59–1.47)^d^	0.759
Change in NIHSS score at 24 h, median (IQR)	-4(−9 to 0)	-6 (−10 to −1)	1.17(−0.36–2.70)^e^	0.135	0.49(−0.97–1.94)	0.510	0.37(−1.24–1.98)	0.654
Change in NIHSS score at 7 days, median (IQR)	−8(−13to −4)	−8 (−13 to −1)	0.96(−0.75–2.67)^e^	0.272	0.32(−1.31–1.95)	0.703	0.06(−1.74–1.86)	0.946
Puncture to recanalization/end of procedure time(IQR)	85(52–129)	90(57–129)	−1.82(−12.05–8.40)^e^	0.726	−8.81(−19.15–1.54)^e^	0.095	−8.99(−19.65–1.68)^e^	0.099
MT PASSES(*n*,%)	1(1–2)	1(1–3)	−0.19(−0.45–0.06)^e^	0.134	−0.02(−0.28–0.24)	0.865	−0.05(−0.32–0.22)	0.722
Successful recanalization at final angiogram^f^	1,374(89.05)	135(91.2)	1.28(0.71–2.31)	0.417	1.34(0.72–2.48)	0.355	1.25(0.68–2.31)	0.478
**Safety outcomes**								
Any ICH within 24 h^g^	325(21.81)	37(25.3)	1.22(0.82–1.80)	0.327	0.89(0.58–1.36)	0.596	0.93(0.61–1.41)	0.724
Symptomatic ICH within 24 h^g^	103(6.97)	11(7.6)	1.10(0.57–2.09)	0.783	0.90(0.46–1.79)	0.767	0.98(0.50–1.93)	0.954
Incidence of intraoperative distal embolism or ectopic embolism	75(4.86)	10(6.8)	1.42(0.72–2.81)	0.316	0.99(0.47–2.10)	0.987	1.00(0.48–2.10)	0.996
Intraoperative complications	1,337(86.65)	126(85.14)	0.88(0.55–1.42)	0.607	1.08(0.65–1.80)	0.765	1.05(0.63–1.74)	0.857

Data are shown as the event number/total number (%), unless otherwise indicated. <80 group was used as the reference group in the model. ^a^Adjusted for the baseline variables with a significant difference of *p* < 0.05 between both groups, including Male sex, History of coronary heart disease, History of atrial fibrillation, History of stroke,Cigarette smoking, NIHSS score, Underlying ICAD, Stroke subtype by TOAST criteria,Balloon angioplasty, Stenting, Intra-procedural use of GPIIb/IIIa, Onset-to-puncture time. ^b^Adjusted for the propensity score. ^c^The common OR values were calculated using an ordinal logistic regression model and indicated the odds of improvement of 1 point on the mRS at 90 days. ^d^The OR values were calculated using a binary logistic regression model. ^e^The β-coefficients were calculated using a generalized linear model. ^f^Defined as mTICI of 2b-3. ^g^According to the Heidelberg Bleeding Classification. CI, confidence interval; ICH, intracranial hemorrhage; IQR, interquartile range; mRS, modified Rankin Scale; mTICI, modified Thrombolysis in Cerebral Infarction; OR, odds ratio; TOAST, Trial of ORG 10172 in Acute Stroke Treatment.

### Subgroup analysis

**Figure 3:**
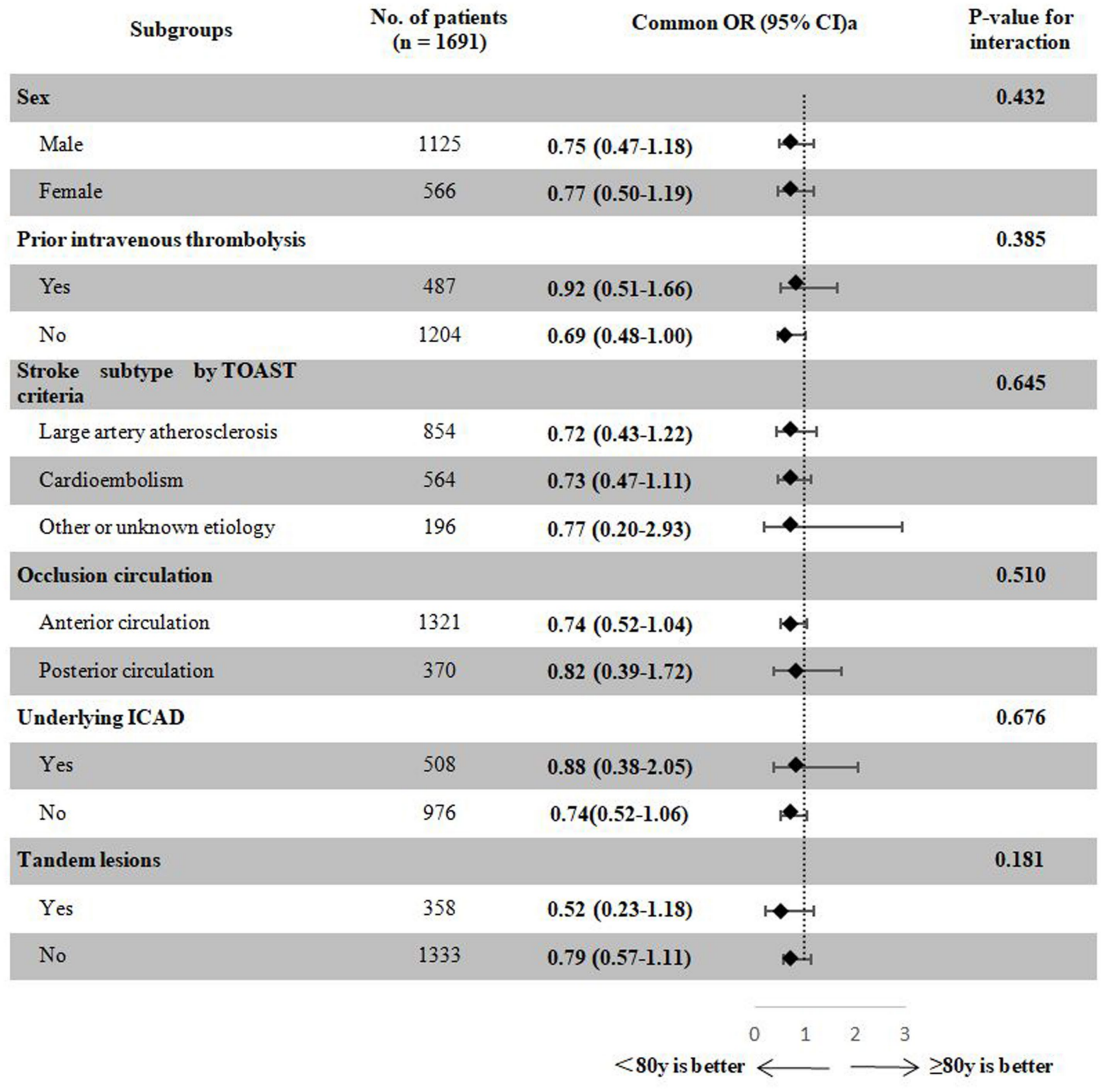
Treatment effects on the primary outcome according to exploratory subgroups. ^a^Adjusted for the propensity score. ^b^A total of 207 patients with undetermined ICAD were excluded. CI, confidence interval; ICAD, intracranial atherosclerotic disease; OR, odds ratio; TOAST, Trial of ORG 10172 in Acute Stroke Treatment.

As shown in Figure 3, similar treatment effect sizes on the primary outcome were observed in the studied subgroups stratified by sex, prior intravenous thrombolysis, stroke subtype according to TOAST criteria, occlusion circulation, underlying ICAD, and tandem lesions (P for interactions > 0.20).

## Discussion

This subgroup analysis of patients with large vessel occlusion (LVO) from the ANGEL-ACT registry found no significant differences between the older and younger populations at 90 days after EVT in terms of safety outcomes. Nevertheless, older adults had a lower rate of moderate functional independence (mRS 0–3) when compared with the younger population. A significantly higher incidence of coronary heart disease, atrial fibrillation history, and higher NIHSS on admission may account for this finding.

The benefits of endovascular therapy (EVT) in older adults with AIS with LVO remain controversial (12–14). A recent meta-analysis conducted by the HERMES collaborators demonstrated that older adults aged ≥ 80 years may benefit more from EVT than conservative treatment (4). However, the results of this study should also be interpreted cautiously, owing to the relatively small number of older adults in this study. Moreover, the elderly typically have more comorbid conditions; therefore, strict patient selection is required. EVT should be performed only in patients who qualify as a registry study based on Solitaire FR stent thrombectomy in North American patients with acute ischemic stroke showed that older age was an independent factor contributing to poor prognosis and high morbidity and mortality rates for mechanical thrombectomy compared with the younger age group (5). This result was supported by the previous relevant studies (6, 15–17) and the recent endovascular Treatment for Acute Ischemic Stroke in the Netherlands (MR CLEAN) Registry study (18).

Our study showed a more neutral result, which is consistent with a previously reported study (19). The rates of excellent outcomes and functional independence among older adults who underwent EVT were comparable to those among younger patients. The fact that there was a higher rate of successful recanalization despite a statistically insignificant difference between the groups may be attributed to this neutral outcome (91.2% vs. 89.1%, *p* = 0.417). Furthermore, shorter onset-to-recanalization times in the elderly group may result in less expansion of the infarct (20). Currently, there is a lack of information in the literature as all the research has been conducted on Western populations. To our knowledge, this study represents the first analysis of real-world multicenter data examining age disparities in EVT in the Chinese population. This result also raises the possibility that social or cultural factors and patient eligibility may have contributed to these differences.

Furthermore, there was no significant difference between the younger and older adults regarding 90-day mortality. However, an age < 80 years was associated with a higher rate of moderate functional independence (OR 0.64, 95% CI 0.44–0.94, *p* = 0.022). The presence of atrial fibrillation and cardiogenic embolism was significantly higher in the elderly than in the younger age group, which might explain this finding. The results of our study are in accordance with previous findings, which reported that cardiogenic embolisms have a less favorable prognosis than large atherosclerotic occlusions due to a lack of collateral circulation (21). This finding might partially explain the lower NIHSS scores at admission in the younger population.

Despite our result showing that EVT is relatively safe in older age, performing early cerebral hemodynamic assessments in elderly patients before and after MT, including transcranial Doppler ultrasound and cerebral perfusion imaging, may reduce the risk of hyperperfusion injury and intracranial hemorrhage. Blood pressure management after recanalization requires further research and may be related to the degree of reperfusion and infarct volume. Moreover, postoperative care is imperative, and patients should be treated using a multidisciplinary approach. A large number of prospective clinical trials are required to address this issue.

A major strength of our study is the large sample size. In addition, since the data were drawn from a prospective national registry of consecutive patients, they may well be representative of actual clinical practice with regard to EVT. However, this study had several limitations. First, despite conventional multivariable analysis and adjustment of the propensity score, this study was not a randomized trial and, therefore, may have resulted in a selection bias. Second, our unscheduled subgroup analysis could not reach any firm conclusions; selective biases need to be considered, and the corresponding results should be interpreted with caution. Third, although these patients were all treated according to the guidelines for EVT, their surgical strategies and postoperative management differed between the hospitals. This may have affected the results to some extent. Furthermore, rehabilitation and psychosocial factors may have a more significant influence on stroke recovery in older adults than in younger adults, but their impact on AIS LVO post-EVT outcomes remains to be explored. Finally, the present study was conducted in the Chinese population; thus, this finding cannot be easily extrapolated to other populations.

## Conclusion

Our results showed that there was no significant difference in the safety and efficacy outcomes (mRS 0–1 and mRS 0–2) between older adults and younger patients who underwent mechanical thrombectomy. However, older adults had a lower rate of moderate functional independence (mRS 0–3) when compared with the younger population. These findings emphasize the need to develop stricter selection criteria for older adults. However, further randomized clinical trials are required to verify this finding.

## Data availability statement

The raw data supporting the conclusions of this article will be made available by the authors, without undue reservation.

## Ethics statement

The studies involving human participants were reviewed and approved by NCT03370939. The patients/participants provided their written informed consent to participate in this study.

## Author contributions

ZM supervised and performed quality control for the study. AW performed the statistical analysis. BH, DS, and Raynald acquired the data and wrote the manuscript with input from BJ, XT, AW, DM, FG, NM, and ZM. All authors contributed to the article and approved the submitted version.

## Funding

This work was supported by the National Key Research and Development Program of China, Grant Number 2016YFC1301500 and China Postdoctoral Science Foundation, grant number 2020-YJ-008.

## Conflict of interest

The authors declare that the research was conducted in the absence of any commercial or financial relationships that could be construed as a potential conflict of interest.

## Publisher’s note

All claims expressed in this article are solely those of the authors and do not necessarily represent those of their affiliated organizations, or those of the publisher, the editors and the reviewers. Any product that may be evaluated in this article, or claim that may be made by its manufacturer, is not guaranteed or endorsed by the publisher.
